# Supplementary material for: Oncosis is the predominant type of cell death in rhabdomyolysis following exertional heat stroke
Source: PLoS One. 2025 Mar 25;20(3):e0308586. doi: 10.1371/journal.pone.0308586 (PMC11936275; doi:10.1371/journal.pone.0308586)
Supplement: Attachment — Submitted filename: Response_to_Reviewers_auresp_2.docx [file pone.0308586.s003.docx]

****Response to Reviewers****

We appreciate the careful review and constructive feedback provided by the reviewers. We have carefully considered all the comments and made revisions accordingly. Below is a detailed response to each of the points raised:

****Absteact:****

**Comment 1:** The abstract is rich in data but dense, making it difficult for readers to quickly grasp the key findings and implications. Simplify and organize the content for better flow.

**Reviewer #2:** Clarity and Readability: The abstract is rich in data but dense, making it difficult for readers to quickly grasp the key findings and implications. Simplify and organize the content for better flow.Consistency: There are inconsistencies in spacing (e.g., missing spaces after commas) and formatting (e.g., inconsistent use of abbreviations). Ensure uniformity throughout.
Grammar and Syntax: Certain phrases could be reworded for grammatical correctness and conciseness.
Structure: The abstract lacks a clear division between the background, objectives, methods, results, and conclusions. These sections should be clearly identifiable for better readability.

**Response:** We have revised the abstract to ensure a more organized structure and simplified language. The key findings and implications are now presented in a clearer and more concise manner. We have broken down the dense content into smaller, digestible segments and added transitions to improve the flow.Here is the revised Abstract based on your editor's feedback:

*****Abstract*****

*****Background:***** *Rhabdomyolysis (RM), particularly heat exhaustion-associated rhabdomyolysis (ehsRM), is a significant clinical issue associated with high mortality and healthcare costs. However, the cellular death mechanisms remain incompletely understood. Oncosis, a form of passive cell death distinct from apoptosis, is characterized by cell swelling and triggered by ATP depletion. Additionally, porimin, a specific biomarker, can uniquely identify oncosis. This study aims to investigate the role and mechanisms of oncosis in both in vitro and in vivo models of ehsRM.*

*****Objective:***** *This study aims to investigate the role and mechanisms of oncosis in both in vitro and in vivo models of ehsRM.*

*****Methods:***** *In the in vitro study, 6-8-week-old male rats were subjected to treadmill exercise at an ambient temperature of (39.5 ± 0.5) °C and relative humidity of 50%-60%, at a speed of 15 meters per minute until their core body temperature (Tc) reached 43.0 °C to establish a heatstroke animal model. Skeletal muscle and blood samples from the gastrocnemius were collected for cytokine, biochemical, and histopathological analyses. Pathological findings revealed decreased muscle fiber density, structural disarray, swelling, degeneration, and hemorrhage. Ultrastructural analysis showed cell swelling, structural disarray, cytoplasmic vacuolation, mitochondrial swelling and degeneration, loss of cristae, and nuclear degeneration, indicating myocyte swelling and necrosis. Porimin, CytC, Bax, and caspase-1 expression increased, while Bcl-2 expression decreased. JC-1 staining indicated a decline in mitochondrial membrane potential and dysfunction. ATP levels decreased, and reactive oxygen species (ROS) production increased.*

*In the in vivo study, HSKMC cells were subjected to 4 hours of heat shock at 43°C to establish a heatstroke-induced rhabdomyolysis cell model. Electron microscopy revealed cell swelling, cytoplasmic vacuolation, mitochondrial swelling and degeneration, and nuclear swelling; late-stage (necrotic-like death) was characterized by nucleolar dissolution, nuclear fragmentation, chromatin condensation, and collapse of cytoplasmic structures. After 24 hours post-modeling, the proportion of double-positive cells (porimin+/PI+) and ROS levels significantly increased, as did porimin expression, while mitochondrial membrane potential and ATP levels significantly decreased. The proportion of Annexin V+/PI+ double-positive cells and caspase-3 levels showed no significant changes.*

*****Results:***** *In both in vitro and in vivo studies, oncosis played a crucial role in ehsRM. Pathological and ultrastructural analyses demonstrated cell swelling, structural disarray, mitochondrial damage, and nuclear degeneration. Porimin, CytC, Bax, and caspase-1 expression increased, while Bcl-2 expression decreased. ATP levels decreased, and ROS production increased. In the in vivo study, the proportion of porimin+/PI+ double-positive cells and ROS levels significantly increased, while mitochondrial membrane potential and ATP levels significantly decreased. The proportion of Annexin V+/PI+ double-positive cells and caspase-3 levels showed no significant changes.*

*****Conclusion:***** *Oncosis is predominant in ehsRM, involving mitochondrial dysfunction, ATP depletion, and oxidative stress.*

This version of the Abstract addresses the clarity, readability, consistency, grammar, syntax, and structure as suggested by the editor.

****Consistency:****

**Comment:** There are inconsistencies in spacing (e.g., missing spaces after commas) and formatting (e.g., inconsistent use of abbreviations). Ensure uniformity throughout.

**Response:** We have reviewed the entire manuscript and standardized the spacing, punctuation, and abbreviation usage. We have ensured consistent formatting throughout the document to enhance readability and professionalism.

****Introduction:****

Comment 1: The manuscript effectively introduces exertional heat stroke (EHS) and rhabdomyolysis (RM), but the research hypothesis could be stated more clearly.

Response: We have clarified the research hypothesis in the introduction to make it more explicit. Here is the refined version to ensure clarity and flow:

*"In this study, we hypothesize that oncosis plays a pivotal role in the development of rhabdomyolysis (RM) in exertional heat stroke (EHS). To validate this hypothesis, we first constructed an EHS-RM rat model and observed that the mechanisms underlying this condition include apoptosis, oxidative stress, and oncosis. Specifically, we hypothesized that oncosis is a key contributor to the progression of rhabdomyolysis.*

*To further investigate, we established a heatstroke-induced rhabdomyolysis model using HSKMC cells. Using Western Blot and RT-qPCR, we detected the upregulation of porimin, a specific marker for oncosis, at both the protein and mRNA levels. Additionally, flow cytometry analysis revealed an increased rate of porimin(+)/PI(+) cells, indicating enhanced oncosis.*

*Our results demonstrate that in EHS, the primary mode of skeletal muscle cell death is oncosis rather than apoptosis. Furthermore, we found that oncosis in skeletal muscle cells is closely associated with reactive oxygen species (ROS) production, mitochondrial membrane potential damage, and ATP depletion. These findings provide new insights into the cellular mechanisms of EHS-induced rhabdomyolysis and suggest potential therapeutic targets for managing this life-threatening condition."*

The revised hypothesis is now clearly stated and directly linked to the objectives of the study.

****Materials and Methods:****

**Comment:** Detailed descriptions of the experimental design, including EHS modeling and assays, are provided. However, statistical methods could be elaborated for transparency.

**Response:** We have expanded the description of the statistical methods used in the study to provide greater transparency as follow.

*Experimental data are presented as means ± SD and analyzed with unpaired two-tailed t-tests for pairwise comparisons. For non-normal distributions, medians (IQR) are reported and analyzed with the Wilcoxon rank-sum test. Repeated measures are analyzed using repeated measures ANOVA. Survival rates are analyzed using the Kaplan-Meier method and log-rank test. All analyses are performed in GraphPad Prism (version 8.3.0, San Diego, CA, USA) with significance at P < 0.05.*

****Results:****

**Comment:** Results are well-organized and supported by figures and tables. However, the results section could benefit from a more narrative flow linking findings to the study objectives.

**Response:** We have enhanced the narrative flow of the results section by explicitly linking the findings to the study objectives. This helps readers understand how each result contributes to the overall research question.

****Discussion:****

**Comment:** The discussion highlights the significance of oncosis but should better integrate findings with existing literature to emphasize novelty.

**Response:** Thank you for your valuable feedback and suggestions. Below is the revised section incorporating the innovations as per your advice:

*****Innovations*****

*****Improved Cell Modeling Conditions:***** *We refined the heat shock conditions to a 43°C water bath for 4 hours, effectively creating a model of heat stroke-induced rhabdomyolysis in muscle cells. This approach is more efficient and stable compared to the previous method of a 43°C incubator for 2 hours.*

*****In Vivo Validation:***** *Currently, there is limited research on tumor dissolution in heat stroke-related rhabdomyolysis both domestically and internationally. Our in vivo experiments validate the role of tumor dissolution, offering new directions for research and treatment of rhabdomyolysis.*

*****Unified Detection Methods:***** *There are varying methods to verify tumor dissolution in cells, lacking a standardized approach. Based on Porimin protein being a recognized marker for tumor dissolution, we propose that Porimin+/PI+ double-positive cells indicate tumor dissolution. By using flow cytometry to measure the proportion of Porimin+/PI+ double-positive cells and comparing it with Annexin V+/PI+ double-positive cells, we found that tumor dissolution, rather than apoptosis, is the primary cause of muscle fiber degeneration and necrosis in heat shock injury.*

We believe these innovations significantly enhance the novelty and robustness of our study. Your comments have been instrumental in guiding this improvement. Thank you once again for your insightful feedback.

****Conclusion:****

**Comment:** The conclusion is concise but would benefit from a clearer statement of implications and future directions.

**Response:** Thank you for your valuable feedback. Below is the revised conclusion incorporating the implications and future directions as per your advice:

*****Conclusion*****

*This study constructed rat models of exertional heatstroke with rhabdomyolysis both in vivo and in vitro to investigate cell death mechanisms. In vivo, oncosis was identified as a key factor, linked to mitochondrial dysfunction and ATP depletion due to ROS damage and UCP-2 activity. In vitro, oncosis, not apoptosis, was confirmed as the primary cell death mechanism, driven by increased ROS, mitochondrial damage, and ATP depletion.*

*This study shows that ATP depletion and mitochondrial dysfunction are critical in muscle cell death during rhabdomyolysis, with oncosis being the main form of cell death. Future research should focus on the signaling pathways causing ATP depletion and explore therapies to enhance ATP levels or protect mitochondria. These findings may lead to new treatments for rhabdomyolysis and related conditions.*

We appreciate your guidance in enhancing the clarity and impact of our conclusions. Thank you once again for your insightful feedback.

****References****

We appreciate your valuable feedback regarding our manuscript. We have thoroughly reviewed our reference list as per your instructions. After careful consideration, we have decided to remove Reference 19 (Tang QF, et al., Bangladesh Journal of Pharmacology, 2018) since it has been retracted.

Regarding Reference 21 (Chun-Li D, et al., Med J China PLA, 2020), although it is a published article in a Chinese journal, we believe it is crucial to our discussion and there are no suitable replacements. Therefore, we have decided to retain this reference and hope you will provide us with further guidance on how to handle this situation appropriately.

Sincerely,
Yi Shan

Department of Emergency Medicine, Sixth Medical Center, PLA General Hospital
