# Supplementary material for: Oncosis is the predominant type of cell death in rhabdomyolysis following exertional heat stroke
Source: PLoS One. 2025 Mar 25;20(3):e0308586. doi: 10.1371/journal.pone.0308586 (PMC11936275; doi:10.1371/journal.pone.0308586)
Supplement: Attachment — Submitted filename: Response to Reviewers.docx [file pone.0308586.s002.docx]

Reviewer #1:

The authors find that EHS rhabdomyolysis is associated with oncosis. However, there are major concerns that reduce the reliability of the study results and enthusiasm for the work. First, the novelty of this work is unclear. In the introduction, the authors suggested that evidence already exists "the mechanism and morphological changes of rhabdo exhibit striking similarities to those observed in oncosis". Second, is this a study of EHS or rhabdo? It is known that only a small portion of EHS patients are associated with rhabdo. It seemed that all the RM rats suffered a heat stroke. Finally, my biggest concern is an insufficient sample size that supports the conclusions. As described, this study included 12 rats, and they were divided into CN and EHS groups (6 per group). Fig 1B indicates that 4 of the 6 rats were lost before 6 hr post-EHS. The animal samples were obtained 6 hrs after EHS. So only 2 rats were included in the EHS group for comparison.

Dear Editor,Thank you for your valuable feedback and for bringing these important points to our attention. Below are detailed responses to address each of your concerns:

Novelty of the Work: We understand that the novelty of our work may not be immediately clear from the current introduction. To clarify the innovative aspects of our research, we will expand on the existing literature to highlight the unique contributions of our study.

First of all, in terms of cell modeling, previous studies mainly used heat shock in 43℃ incubator for 2-hour modeling. This experiment repeated the modeling condition and extended the heat shock time to 4 hours, but the damage of human skeletal muscle cells was still relatively light. Considering that the efficiency of heat conduction is higher than that of heat radiation, this study improved the HSKMC thermal shock conditions and used 43℃ water bath for 4 hours to construct the rhabdomyolysis cell model of thermal radiation disease, which can be more efficient and stable.

Secondly, there are few studies on the role of swelling and death in rhabdomyolysis related to thermal radiation disease at home and abroad, and only a few in vitro studies have been conducted. On the basis of previous in vitro experiments, this study verified the role of swelling and death in ehsRM in vivo, which is a new direction for the research and treatment of rhabdomyolysis.

Finally, the current domestic and foreign research on the oncosis is still insufficient, especially in how to verify the cell oncosis means are different, there is no unified standard, the current recognized method is the detection of electron microscopy ultramicrostructure and porimin protein. Flow cytometry has also been used in many studies of dilatation, but mostly with Annexin V/PI double staining, and opinions are divided. Some scholars believe that the dilatation cells are (Annexin V+/PI+) Shuangyang cells, and some scholars believe that the Annexin V+/PI- cells are (Annexin V+/PI-). Some scholars believe that Annexin V-/PI+ is dilatation-like necrotic cells. It is well known that Annexin V-FITC/PI double staining flow cytometry is a classic apoptosis detection method, and it is considered that (Annexin V+/PI-) cells are premature, (Annexin V+/PI+) double positive cells are late, and (Annexin V-/PI+) cells are necrotic cells. In view of the conflicting views on the identification of dilatatory cells by Annexin V-FITC /PI double staining flow cytometry and the fact that porimin protein is a recognized dilatatory cell membrane protein, we consider (porimin+/PI+) double positive cells to be dilatatory cells. Flow cytometry (porimin+/PI+) and Annexin V+/PI+ double Yang cell rates were used to compare the effects of dilatation and apoptosis on rhabdomyolysis in thermal radiation disease. The results showed that dilatation, rather than apoptosis, was the main form of death in rhabdomyolysis in thermal radiation disease.

****2.Clarity on Study Focus****: We acknowledge that the distinction between EHS and rhabdomyolysis might not be entirely clear in our manuscript. To address this, we will explicitly state that our primary objective is to investigate the association between EHS and rhabdomyolysis, particularly focusing on the subset of EHS patients who develop rhabdomyolysis. We will also provide additional context to explain why our model uses rats suffering from both EHS and rhabdomyolysis. This will help readers better understand the rationale behind our experimental design.

****3.Sample Size Concerns****: We recognize that the sample size of 12 rats, with only 2 surviving in the EHS group at the time of data collection, is indeed a limitation. To strengthen our conclusions, First of all, although only 2 rats in the EHS group survived at 6h, we included the gastrocnemius of all rats in the study, so the sample size of our study was 6, and we found that these gastrocnemius also had rhabdomyolysis.secondly,we plan to conduct additional experiments with a larger cohort of animals. Specifically, we aim to increase the number of rats in each group to at least 10-15, which would provide more robust statistical power. Additionally, we will perform survival analysis to account for the loss of animals over time and ensure that our conclusions are based on a more comprehensive dataset.

Abstract:ehsRM was defined twice. The conclusions are confusing.

Thank you for your careful review of our paper and your valuable comments. We apologize for your point that ehsRM was defined twice, which led to confusion in the conclusion section. After careful examination, we confirmed that this was due to an oversight that occurred during the writing process. To ensure the clarity and accuracy of the paper, we have re-examined the relevant sections and unified definition: We remove duplicate definitions and keep only one clear and consistent definition as”rhabdomyolysis associated with heat exhaustion (ehsRM)”.

Introduction:Some statements are misleading. For example, there is no evidence for "Oncosis, a form of porimin-dependent non-programmed cell death, is caspase independent.".

Thank you for your thorough review and valuable feedback on our paper. Regarding the comment that "certain statements are misleading, such as 'necrosis (oncosis), a non-programmed form of cell death dependent on osmotic proteins, is caspase-independent' lacks evidential support," we respectfully disagree with this point. We have reviewed additional literature and would like to incorporate more supporting references in our revised manuscript.

We believe that necrosis, often referred to as oncosis, can indeed be considered a type of non-programmed cell death that is not directly dependent on caspases under certain conditions. However, it is important to note that the exact mechanisms and regulatory pathways involved in necrosis are still being actively researched. To support this perspective, we have included several key references below:

Oncosis, a form of porimin-dependent**^[1-3]^** non-programmed cell death**^[4]^**, is caspase independent**^[5]^**.".

**1.[Reference 1 to 3]** - These studies discusse the role of porimin in oncosis and has been shown that oncosis, like apoptosis, can be triggered by the activation of cell surface receptors, such as the PORIMIN (pre-oncosis receptor induced membrane injury) receptor.

**2.[Reference 4]** - This paper explores the diverse forms of necrotic cell death and provides evidence that necrosis can occur independently of caspase activation.

**3.[Reference 5]** - This review article synthesizes findings from multiple studies and discusse the role of osmotic stress in inducing necrosis and highlights the independence from caspase activation.

By incorporating these references, we aim to provide a more comprehensive understanding of the mechanisms involved in oncosis and address the concerns raised in your feedback.

Results:In addition to mean/SD, individual values should be displayed in all the bar graphs

Thank you for your valuable suggestions. I have marked the changes according to your feedback in the figure.If you need any further adjustments or additional help, feel free to let me know!

3.1: How do you know "onset"? There are no additional data other than that at 6 hr post-EHS. Should this be Table 3? why did CN rats lose weight as well?

How do you know "onset"? :The onset is defined based on the behavior, mental state, and core body temperature of the rats: it is characterized by exhaustion with a core body temperature of 43°C or higher. Exhaustion is specifically manifested as follows: under painless prodding, the rats are unable to stand, have a movement score of 2 (able to perform full range joint movements such as righting reflex (+), limbs flexed, but unable to stand), wet fur, convulsions, lethargy, and even light coma. Finally, we observe that the levels of CK and Mb exceed five times the normal values, and pathological examination shows swelling, structural disarray, and even rupture of striated muscle.

**Table .4 Rat exercise rating criteria（Revised with reference to Lovett grading method）**

| **Rating** | **Motion condition** |
| --- | --- |
| Lv.0 | Stimulation does not escape contraction |
| Lv.1 | There is slight contraction, but it does not cause joint movement, such as: righting reflex (-), cannot raise its head, and the limbs are straight |
| Lv.2 | In the state of weight loss can perform full range of joint movement, such as: righting (+), limbs flexion, but unable to stand |
| Lv.3 | Can resist gravity for full range of joint movement, but can not resist resistance, such as: creeping forward |
| Lv.4 | Can resist gravity, resistance to certain resistance movement, such as: can stand on four legs walking, independent foraging |
| Lv.5 | Can resist gravity, resistance movement, such as: hind legs standing, climbing |

There are no additional data other than that at 6 hr post-EHS. :Prior to this study, a preliminary experiment was conducted to compare the change trend of body temperature, behavior, inflammatory factors, creatine kinase and myoglobin within 24 hours of the two groups of rats. After successful modeling, the rats were removed from the high-temperature simulated environment, recovered naturally at room temperature, and continued to monitor their body temperature intermittently. As shown in Figure 2-3, the rat's anal temperature decreased rapidly, dropping to (35.97 ± 1.22)℃ within 1-2 hours, and then the body temperature gradually recovered, and the anal temperature gradually recovered to (37.30 ± 0.71)℃ about 6 hours later.，It was found that the TNF-α, IL-1β, IL-6, CK and MB indexes of the EHS group showed a significant increase trend compared with that of the CN group within 6 hours after the successful modeling. The peak value was observed at T6h. Subsequently, the levels of inflammatory factors in EHS group were gradually decreased. Based on this finding, we selected 6 hours after the occurrence of thermal radiation disease as the time point for collecting research samples.

why did CN rats lose weight as well?：In order to reduce the error, the CN group rats were deprived of water during the modeling period. During the modeling period, the rats would urinate and poop, and could lose water invisibly with breathing, so the CN group rats would also have a slight weight loss.

3.2: oxidative stress. There are no ROS makers in Fig 2

Thank you for your valuable feedback. We appreciate your attention to detail. The ROS (reactive oxygen species) detection results are indeed presented in Figure 5. To ensure clarity and avoid any confusion, we will modify the text in Section 3.2 to explicitly mention that the ROS detection results are shown in Figure 5. This will help readers easily locate and understand the relevant data. Please let us know if this adjustment meets your expectations.

Reviewer #2:

This manuscript investigates the role of oncosis as the primary mechanism of cell death in exertional heat stroke-associated rhabdomyolysis (ehsRM). While the study presents a compelling argument supported by in vitro and in vivo experiments, there are several areas that could be improved or further elaborated. Below are specific comments
1.Mechanistic Link Between Mitochondrial Dysfunction and Oncosis: While the study documents mitochondrial damage and links it to oncosis, the exact molecular mechanisms connecting mitochondrial dysfunction and oncosis could be elaborated further. How mitochondrial swelling directly contributes to oncosis versus other forms of necrosis could be clarified, especially in relation to the release of DAMPs (damage-associated molecular patterns) or mitochondrial permeability transition (MPT).

Thank you for your insightful feedback. Our study indeed documents the significant association between mitochondrial dysfunction and oncosis in skeletal muscle cells, particularly under conditions of heat stress and ATP depletion. We propose that this leads to increased ROS production and enhanced release of CytC, thereby exacerbating mitochondrial dysfunction. However, the exact molecular mechanisms underlying this process require further investigation, and this is a key focus of our ongoing research.

While we acknowledge the potential roles of damage-associated molecular patterns (DAMPs) and mitochondrial permeability transition (MPT) in linking mitochondrial dysfunction to oncosis, our current study primarily aims to validate the critical role of oncosis in heat stroke-induced rhabdomyolysis. Given the scope and objectives of our present study, we have chosen to concentrate on demonstrating the importance of oncosis rather than delving deeply into the specific mechanisms involving DAMPs or MPT.

We recognize the importance of these mechanisms and plan to explore them in future studies. For now, we believe that establishing the central role of oncosis in the context of heat stroke-related rhabdomyolysis is a significant step forward. We appreciate your suggestions and will consider addressing these aspects in our future work.

This response aims to clearly articulate the focus and scope of the current study while acknowledging the broader implications and future research directions related to DAMPs and MPT.

Involvement of Mitochondrial Dynamics: Mitochondrial dynamics (fusion, fission, mitophagy) are critical for maintaining mitochondrial integrity, especially under stress conditions. The manuscript does not address whether there are any disruptions in mitochondrial dynamics that could contribute to the observed mitochondrial swelling and dysfunction. Including markers of fusion (e.g., MFN2, OPA1) and fission (e.g., DRP1) could provide a more comprehensive understanding of how mitochondria fail during ehsRM.

Thank you for your valuable feedback and suggestions. We appreciate the opportunity to clarify and enhance our manuscript based on your insights.

We agree that mitochondrial dynamics, including fusion, fission, and mitophagy, play crucial roles in maintaining mitochondrial integrity, especially under stress conditions such as those encountered in exertional heat stroke-related rhabdomyolysis (ehsRM). However, our current study primarily focuses on the direct effects of mitochondrial dysfunction and its consequences, specifically the role of mitochondrial swelling and the subsequent oncosis.

To address your comments more comprehensively, we acknowledge the importance of investigating mitochondrial dynamics in future studies. Specifically, evaluating markers of fusion (such as MFN2 and OPA1) and fission (such as DRP1) would indeed provide a more detailed understanding of the mechanisms underlying mitochondrial failure in ehsRM.

We propose incorporating these markers in our future research to elucidate the interplay between mitochondrial dynamics and the observed mitochondrial swelling and dysfunction. This additional data will strengthen our understanding of the pathological processes involved in ehsRM and contribute to a more complete picture of the disease mechanism.

Thank you once again for your constructive feedback. We look forward to incorporating these enhancements in our future work.

3.Incomplete Exploration of ROS-Mediated Mitochondrial Damage: Although the study demonstrates increased ROS levels and mitochondrial dysfunction, it does not fully explore the interplay between ROS production and mitochondrial damage. Mitochondria are both a source and target of ROS. An investigation into whether mitochondrial ROS production exacerbates the observed dysfunction would be valuable, possibly by using mitochondrial-targeted antioxidants in additional experiments.

Thank you for your valuable comments and suggestions. We welcome the opportunity to further improve our manuscript.

We agree with your observation that while our study suggests elevated ROS levels and mitochondrial dysfunction, it does not adequately explore the interaction between ROS production and mitochondrial damage. In fact, mitochondrial damage is both a source and a result of ROS, and understanding this complex relationship is critical for a comprehensive analysis of mitochondrial dysfunction in exercise-induced heatstroke associated rhabdomyolysis (ehsRM).

However, the purpose of observing mitochondrial changes in this study is to distinguish them from apoptosis, and the purpose of this study is mainly to verify the role of oncosis rather than study the mechanism of oncosis. The occurrence of oncosis induced by the mutual causal relationship between ros level rise and mitochondrial damage is also the preliminary assumption of the mechanism of oncosis in this study, and we hope that the next research can further explore the mechanism of oncosis.

4.Mitochondrial Bioenergetics: The study primarily measures ATP levels, but a more detailed analysis of mitochondrial bioenergetics, including the activity of key respiratory chain complexes (e.g., Complex I-IV), would strengthen the conclusions regarding mitochondrial dysfunction.

Thank you for your valuable feedback and suggestion to enhance our study by including a more detailed analysis of mitochondrial bioenergetics, particularly the activities of key respiratory chain complexes such as Complexes I-IV. We appreciate your insight and agree that this additional information could provide deeper insights into mitochondrial dysfunction.

However, We believe that the primary focus on ATP levels has already provided significant evidence for our conclusions, and while we acknowledge the potential benefits of the proposed analysis, it is beyond the scope of what we can accomplish with our present constraints.

We hope to address this limitation in future studies where we can allocate the necessary resources and time to perform a comprehensive assessment of mitochondrial bioenergetics. In the meantime, we have strengthened our discussion to highlight the importance of these additional analyses and how they might further elucidate mitochondrial function and dysfunction.

Thank you once again for your understanding and support.

Recommended Additional Experiments
1.Mitochondrial Dynamics: Assess the expression of proteins involved in mitochondrial fusion (MFN1/2, OPA1) and fission (DRP1, FIS1). This could help elucidate whether altered mitochondrial dynamics contribute to the observed mitochondrial swelling and dysfunction in ehsRM.

Thank you for your insightful suggestion to assess the expression of proteins involved in mitochondrial dynamics, specifically those related to fusion (MFN1/2, OPA1) and fission (DRP1, FIS1). We understand the potential value of this analysis in elucidating the role of altered mitochondrial dynamics in the observed mitochondrial swelling and dysfunction in ehsRM.

However, due to the current constraints of our experimental setup and resource availability, we are unable to include these additional analyses at this time. We believe that the data we have presented on mitochondrial swelling and dysfunction provides a solid foundation for our conclusions, but we recognize the importance of mitochondrial dynamics in this context.

To address this gap, we have discussed the potential contributions of mitochondrial dynamics in our manuscript and highlighted the need for future studies to investigate these aspects. We are committed to expanding our research in this direction in subsequent investigations where we can incorporate these crucial measurements.

We hope you find our response satisfactory and appreciate your understanding of our current limitations.
**2.Mitochondrial Respiration: Perform mitochondrial respirometry to quantitatively assess mitochondrial respiratory function, including oxygen consumption rate (OCR), basal respiration, ATP production, and maximal respiratory capacity in the EHS model.**

Thank you for your valuable suggestion regarding the assessment of mitochondrial respiratory function through mitochondrial respirometry. We appreciate the importance of this technique in providing quantitative data on oxygen consumption rate (OCR), basal respiration, ATP production, and maximal respiratory capacity in the EHS model.

Indeed, monitoring mitochondrial function encompasses a variety of methods, each contributing unique insights into the structural and functional changes associated with mitochondrial dysfunction. Our current study employs several complementary approaches to comprehensively evaluate mitochondrial health:

A.Morphological Observations: Using transmission electron microscopy (TEM) to directly visualize mitochondrial morphology and structure.

B.Reactive Oxygen Species (ROS) Detection: Utilizing the fluorescent probe DCFH-DA to measure intracellular ROS levels.

C.ATP Content Measurement: Employing a luciferase-based assay to quantify intracellular ATP levels.

D.Mitochondrial Membrane Potential (ΔΨm) Assessment: Using JC-1 fluorescence dye to evaluate ΔΨm.

These methods collectively provide a robust framework to elucidate the structural and functional alterations of mitochondria in the context of heat stroke-related rhabdomyolysis. However, we acknowledge that mitochondrial respirometry would further enhance our understanding of mitochondrial respiratory function and its impact on cellular metabolism.

While we are currently constrained by the scope of this study, we agree that incorporating mitochondrial respirometry in future experiments will be essential to fully characterize the underlying mechanisms of mitochondrial dysfunction. We plan to pursue these deeper investigations in subsequent studies.

We hope you find our response satisfactory and thank you for your constructive feedback.

**3.Mitochondrial ROS Scavengers: Introduce mitochondrial-targeted antioxidants to determine whether reducing mitochondrial ROS levels can alleviate mitochondrial damage and oncosis. This could provide a therapeutic angle to prevent or mitigate cell death in ehsRM.**

Thank you for your insightful suggestion regarding the introduction of mitochondrial-targeted antioxidants to investigate their effects on mitigating mitochondrial damage and oncosis.

Our study has observed a strong correlation between reactive oxygen species (ROS) and mitochondrial damage. Numerous studies have demonstrated a bidirectional relationship between ROS and mitochondrial dysfunction, where ROS can exacerbate mitochondrial damage, and conversely, mitochondrial damage can lead to increased ROS production. Importantly, evidence from various studies indicates that mitochondrial ROS scavengers can indeed reduce mitochondrial damage. Furthermore, some research has confirmed that mitochondrial ROS scavengers can alleviate cell swelling and death, which is particularly relevant to our investigation of rhabdomyolysis.

In our current experiment, we aim to focus on validating the relationship between oncosis and rhabdomyolysis, In this experiment, we aim to focus on validating the relationship between oncosis and rhabdomyolysis. We believe that incorporating mitochondrial ROS scavengers into our future experimental design will not only strengthen our understanding of the underlying mechanisms but also provide a potential therapeutic strategy to prevent or mitigate cell death in heat stroke-related rhabdomyolysis.

We appreciate your guidance and look forward to further refining our study based on your valuable suggestions.

**4. Ambiguity in Statistical Presentation: The manuscript does not consistently report statistical significance (e.g., p-values) across experiments. Additionally, error bars on graphs are not always explained (e.g., standard deviation vs. standard error). Ensure that all statistical analyses are clearly reported, with appropriate p-values and error bars labeled. Use more rigorous statistical tests where appropriate.**

Thank you for your feedback. We will revise the manuscript to ensure consistent reporting of statistical significance using p-values. We will also clearly explain the meaning of error bars (standard deviation or standard error) and use more rigorous statistical tests where appropriate.
5. Lack of Functional Outcomes or Behavioral Correlates in Vivo: The manuscript focuses heavily on molecular and structural analysis but lacks a functional or clinical correlate for rhabdomyolysis and EHS severity in the rat model. Behavioral or functional assessments of muscle function (e.g., grip strength, endurance) post-EHS would help translate these findings into clinically relevant outcomes. Include behavioral or physiological assessments in the rat model to assess muscle function after EHS and correlate them with biochemical and histopathological findings.

thank you for your valuable feedback and suggestions. We acknowledge the importance of including functional outcomes and behavioral correlates in our study to better translate our findings into clinically relevant outcomes.

In response to your specific comments, we would like to provide additional context regarding the development and validation of our animal models. Compared to classic heat stroke, exertional heat stroke (EHS) is considered a more severe acute condition, characterized by more severe symptoms, faster progression, and higher incidence and mortality rates [7]. Rhabdomyolysis (RM) is a significant complication in EHS patients, with a mortality rate as high as 50% [7]. However, in experimental conditions, the incidence and mortality rates of RM are lower, which is inconsistent with clinical data. Therefore, developing a good preclinical animal model is crucial for our research.

Previous models described by King et al. [90] had limitations and could not fully reflect the key clinical features of EHS patients. For instance, while creatine kinase (CK) levels were elevated, they were not significantly so, and all mice survived over a 4-day experiment. He et al. [87] designed four different EHS conditions with relatively high temperatures and humidity levels (37.5°C, 65% RH; 37.5°C, 70% RH; 39.5°C, 65% RH; and 39.5°C, 70% RH). Based on a 24-hour survival rate of 50%, they determined that 39.5°C, 65% RH was optimal for EHS modeling. Mice in the EHS group ran at a constant speed of 15 rpm on a treadmill until exhaustion or when their core temperature reached 43°C. This model better reflected the clinical characteristics of EHS, such as CK levels exceeding 10,000 U/L and a 24-hour survival rate of 37.34%.

Han-Ding Mao et al. [88] established an EHS rat model under conditions of (39.5 ± 0.3)°C and (55 ± 5)% RH. Following this, we used similar conditions to create our own model. However, during our experiments, we observed that almost all rats reached a core temperature of 43°C, but some did not reach exhaustion. Moreover, the survival rate of these rats was much higher than 50%. We hypothesize that this discrepancy may be due to differences in the rats' labor tolerance and heat resistance. Therefore, we adopted a criterion where rats must be exhausted and have a core temperature (Tc) ≥ 43°C for the model.

To standardize the criteria for exhaustion, we referred to the human Lovett grading system and made adjustments based on our team's discussions. The final grading criteria are shown in Table.4. The lower the exercise grade, the more severe the exhaustion and the higher the mortality rate. After repeated validations, we confirmed that the exhaustion criterion is when rats cannot move even under non-painful prodding, exhibiting a persistent crawling posture. At this point, the exercise grade is set to 2 (positive righting reflex, limbs flexed but unable to stand), with a core temperature (Tc) ≥ 43°C.

Table .4 Rat exercise rating criteria（Revised with reference to Lovett grading method）

| **Rating** | **Motion condition** |
| --- | --- |
| Lv.0 | Stimulation does not escape contraction |
| Lv.1 | There is slight contraction, but it does not cause joint movement, such as: righting reflex (-), cannot raise its head, and the limbs are straight |
| Lv.2 | In the state of weight loss can perform full range of joint movement, such as: righting (+), limbs flexion, but unable to stand |
| Lv.3 | Can resist gravity for full range of joint movement, but can not resist resistance, such as: creeping forward |
| Lv.4 | Can resist gravity, resistance to certain resistance movement, such as: can stand on four legs walking, independent foraging |
| Lv.5 | Can resist gravity, resistance movement, such as: hind legs standing, climbing |

We believe these revisions will enhance the clinical relevance of our findings and provide a more robust preclinical model for further research.

Thank you again for your insightful comments and suggestions.
**6. Limited Validation of Oncosis Markers: While the study highlights porimin as a marker for oncosis, the absence of multiple independent markers limits the specificity of the conclusions. Porimin alone may not provide conclusive evidence of oncosis as the dominant mechanism, especially given c. Incorporate additional markers (e.g., HMGB1, or others associated with oncosis) and perform colocalization experiments with mitochondrial damage markers to reinforce the claim of oncosis.**

While the current literature reports and confirms porimin protein as a specific marker for oncosis, the existing study still faces limitations due to the absence of multiple independent markers, which restricts the specificity of the conclusions. Porimin alone may not provide conclusive evidence that oncosis is the dominant mechanism, particularly when apoptotic markers such as Bax/Bcl-2 are also present. To strengthen the claim of oncosis, it would be beneficial to incorporate additional markers like HMGB1 and other markers specifically associated with oncosis, and perform colocalization experiments with mitochondrial damage markers.

As the author, we believe that our study has validated porimin protein as a specific marker for oncosis through multiple detection methods including Western Blot (WB), quantitative PCR (qPCR), immunohistochemistry (IHC), and immunofluorescence in both animal and cell models. Additionally, we have improved the flow cytometry method for detecting oncosis. The conventional Annexin V/PI double staining flow cytometry technique is widely used in necrosis studies, but there is inconsistency in its application. Some scholars consider (Annexin V+/PI+) cells as necrotic, while others identify (Annexin V+/PI-) cells as early apoptotic and (Annexin V-/PI+) cells as necrotic-like cells. Given the inconsistent opinions regarding the use of Annexin V-FITC/PI double staining flow cytometry to distinguish oncosis cells, and considering that porimin is a recognized membrane protein of oncosis cells, we propose that (porimin+/PI+) cells represent oncosis cells. We used flow cytometry to detect the rate of (porimin+/PI+) cells and compared it with the rate of (Annexin V+/PI+) cells to contrast the roles of oncosis and apoptosis in heat stroke-induced rhabdomyolysis. Our results show that oncosis, rather than apoptosis, is the primary form of cell death in this condition.

Furthermore, cellular morphology is a critical criterion for determining the mode of cell death. Apoptosis and oncosis are distinct forms of cell death, where apoptosis is an energy-dependent process characterized by cell shrinkage. In contrast, oncosis is caused by energy depletion and exhibits morphological features such as cell swelling, vacuolation, loss of intracellular organelles, disruption of cell membrane integrity, endoplasmic reticulum swelling, mitochondrial swelling, nuclear envelope dispersion, and chromatin condensation. Ultimately, the release of intracellular contents triggers an inflammatory response. Our study combines multiple detection methods for porimin protein, ultrastructural changes observed via electron microscopy, and alterations in mitochondrial structure and function from various perspectives to validate the primary role of oncosis in rhabdomyolysis during heat stroke.

We also acknowledge that the presence of apoptotic markers (such as Bax/Bcl-2) in our in vivo experiments indicates that apoptosis does occur during rhabdomyolysis. This finding aligns with our conclusion in Section 3.2, which states that apoptosis, oxidative stress, oncosis, and other mechanisms are involved in the pathological mechanism of rhabdomyolysis in exertional heat stroke. However, in Section 3.3, we emphasize that oncosis, rather than apoptosis, is the primary form of skeletal muscle cell death.
**7.Unclear Differentiation between Oncosis and Apoptosis: The manuscript claims that oncosis is the predominant form of cell death, but the presence of apoptotic indicators such as Bax and Bcl-2 raises questions. Although caspase-3 levels did not change, the manuscript does not adequately explain why apoptosis, in addition to oncosis, is not more prominent.Conduct time-course experiments to clearly differentiate the dynamics of oncosis and apoptosis. A detailed analysis of caspase-independent apoptotic pathways could be beneficial to clarify this distinction.**

Thank you for your valuable feedback on our manuscript. We acknowledge the need to better differentiate between oncosis and apoptosis.Recent studies have confirmed that in certain pathological processes, oncosis (swelling-induced cell death) and apoptosis can coexist. These two forms of cell death exhibit distinct anatomical distributions; cells in well-perfused areas tend to undergo apoptosis more frequently, while cells in poorly perfused areas are more prone to oncosis. Although both apoptosis and oncosis may be triggered by the same stimuli, their occurrence and development depend on the intensity and duration of these stimuli ^[6]^. Under weak stimuli or short-term effects, apoptosis predominates; however, under strong and sustained stimuli, cells may die through the oncosis pathway ^[7]^. Many reports indicate that the mode of cell death is influenced by ATP levels. When cells face the same stimulus, their internal ATP levels determine whether they will undergo apoptosis or oncosis. If ATP levels are sufficient, cells are more likely to undergo apoptosis smoothly. However, if energy supply within the cell is inadequate, the cell cannot undergo apoptosis smoothly and instead undergoes oncosis. Therefore, factors affecting cellular ATP levels also influence the choice of cell death mechanisms.

Heat stroke, particularly exertional heat stroke (EHS), involves skeletal muscle contraction, which is an energy-consuming process. Intense exercise can lead to ATP deficiency or depletion in exRML patients, causing an increase in intracellular Ca²⁺ levels^[8]^. If ATP levels are insufficient, the activity of Na⁺/K⁺-ATPase decreases, leading to dysfunction of Na⁺/K⁺-ATPase, which in turn results in elevated intracellular Na⁺ levels and triggers oncosis. In this experiment, I extracted mitochondria and used luciferase to detect changes in intracellular ATP levels. The results showed a significant decrease in ATP content in the gastrocnemius muscles of EHS rats and HSKMC cells in the HS group. Therefore, we propose that the mechanism of muscle cell death in skeletal muscle cells undergoing oncosis is primarily due to ATP depletion and mitochondrial damage.

We appreciate your guidance and look forward to incorporating these improvements to strengthen the manuscript.

Reviewer #3:

1. Introduction and discussion section should be improved.
The difference between apoptosis and oncosis should be described in detail. Authors should emphasize the importance of this study? Is it really valuable to discriminate oncosis and apoptosis?

Thank you very much for your valuable feedback and suggestions. We appreciate your comments and are committed to improving our manuscript accordingly. Specifically, we will enhance the introduction and discussion sections to provide a more detailed description of the differences between apoptosis and oncosis. Additionally, we will emphasize the importance and value of this study in discriminating between these two types of cell death.

Here is how we plan to address your concerns:

Detailed Description of Apoptosis and Oncosis: In the introduction, we will clearly define and contrast apoptosis and oncosis. Cell apoptosis and collapse refers to the cellular response to injury prior to cell death. These are mainly distinguished by cell volume and nuclear morphology ^[9]^. In the process of apoptosis, cells contract, multiple cytoplasmic processes or vesicles appear, cell membrane permeability does not increase significantly, nuclear fragmentation, nuclear chromatin condensation is obvious, and finally cell fragmentation into apoptotic bodies ^[10]^. Dilatation is associated with energy expenditure, resulting in impaired membrane ion pumps, cell swelling, endoplasmic reticulum and golgi dilation, mitochondrial swelling, nuclear chromatin clumping, and the formation of organell-free cytoplasmic vesicles or vesicles ^[10, 11]^.Apoptosis refers to the process by which cells stop growing and dividing, relying on cysteine aspartate proteases (caspases), and ultimately leading to the controlled death of the cell without causing damage to the surrounding environment. Unlike apoptosis, an important biochemical event that leads to bloating is the rapid decline of intracellular ATP ([ATP]i) ^[9]^. Interference with ATP synthesis rapidly leads to the inactivation of Na+/K+ -ATPase on the cell membrane, resulting in the increase of [Na+]i and [Cl-]i, accompanied by water inflow, cell swelling, and rapid increase of [Ca2+]i ^[12]^.

**Emphasis on the Importance of this Study and value of Discriminating Between Apoptosis and Oncosis**: This study aims to investigate the pathological manifestations of rhabdomyolysis caused by heatstroke and distinguish between oncosis and apoptosis. Understanding the differences between these two forms of cell death is crucial for advancing the mechanisms underlying rhabdomyolysis, developing effective treatments, and devising preventive strategies. Distinguishing between apoptosis and oncosis is also essential for assessing the severity and prognosis of the disease. These two forms of cell death exhibit distinct anatomical distributions; cells in well-perfused areas tend to undergo apoptosis more frequently, while cells in poorly perfused areas are more prone to oncosis. Although both apoptosis and oncosis may be triggered by the same stimuli, their occurrence and development depend on the intensity and duration of these stimuli [6]. Under weak stimuli or short-term effects, apoptosis predominates; however, under strong and sustained stimuli, cells may die through the oncosis pathway [7]. Moreover, apoptosis typically does not cause significant damage to the surrounding environment, whereas oncosis can lead to the release of cellular contents, triggering inflammatory responses.

We believe that these revisions will significantly enhance the quality and clarity of our manuscript, making it more impactful and relevant to the scientific community.

Thank you once again for your valuable feedback. Please let us know if there are any further adjustments needed.

**2. EHS protocol is stopped when the animals become exhausted. Please give the total duration of protocol for each animal.**
Thank you for your valuable feedback and suggestions. In response to your request to provide the total duration of the EHS protocol for each animal, we have updated our results section and included this information in Table 4, alongside the body temperature and body weight measurements for both the EHS group and the control group,To reduce the bias in the results caused by the modeling time, we took 20 minutes as the modeling time for the CN group.Please find the updated Table 4 below:

****Table 4: Body Temperature, Body Weight, and Duration of EHS Protocol****

| **group** | **Weight**  **(g)** | **Tc0**  **(℃)** | **TcEHS**  **(℃)** | **Percent Weight change (%)** | **Duration of EHS protocol**  **（min）** |
| --- | --- | --- | --- | --- | --- |
| CN | 306.40±13.14 | 38.45±0.36 | 38.55±0.27 | 1.90±0.70 | 20 |
| EHS | 301.80±15.44 | 38.55±0.31 | 43.82±0.57 | 7.87±1.54 | 19.00±3.16 |
| *P* | 0.733 | 0.468 | <0.001 | <0.001 | 0.457 |

We hope these additions clarify the necessary details and enhance the clarity of our study findings. If you have any further questions or require additional information, please do not hesitate to contact us.

**3. Authors have provided sterile water. What about chow? Is it also sterile?**
The authors have provided sterile water. Regarding the chow, all rats were fed with laboratory sterilized feed specifically designed for rats after the EHS onset. This ensures that the diet is also sterile and maintains the sterility of the experimental conditions
**4. Authors put cell lines into a hot water bath. How did they gassed the cells? Did authors also consider the solubility change between 37 and 43 °C.**

Although the cell lines were placed in hot water baths at different temperatures (37°C and 43°C) for the CN group and HS group respectively, the culture bottles contained the same gas mixture to provide adequate oxygen to the cells. While there is a potential bias due to changes in gas solubility between 37°C and 43°C, we do not believe this affects our experimental results. Our study focuses on heat stroke-related rhabdomyolysis, and the thermal stress and possible reduction in oxygen solubility due to temperature changes collectively contribute to establishing a human skeletal muscle cell model relevant to heat stroke-related rhabdomyolysis.
 **5. 30 minutes is a relatively long centrifuge time. Why did authors prefer a long centrifuge time?**
Thank you for bringing this to our attention. There was a typographical error in the manuscript. The correct centrifugation time we used in our experiments was indeed 10 minutes, not 30 minutes. We apologize for any confusion this may have caused.
**6. Authors measured CK. Is it total? Were they able to identify subtypes?**
The measurement of CK (Creatine Kinase) in our study refers to the total CK level. We did not specifically measure or differentiate into its subtypes, such as CK-MM, CK-MB, and CK-BB, due to the limitations in resources and the specific focus of our research. **7. Could authors cite the previous study? The study in which they first made the EHS model.**
Certainly, The EHSmodel of this study benefits and improves with the following research. Specifically, He et al. ^[13]^ designed four relatively high temperature and humidity conditions for the EHS model: (37.5°C, 65% RH; 37.5°C, 70% RH; 39.5°C, 65% RH; and 39.5°C, 70% RH). Based on the criterion of a 24-hour 50% mortality rate, they ultimately determined that the condition of 39.5°C and 65% RH was suitable for EHS modeling. In this model, mice were made to run on a treadmill at a constant speed of 15 rpm/min under these conditions until exhaustion or when their core body temperature reached 43°C. This model more closely aligns with the clinical characteristics of EHS, where creatine kinase levels typically exceed 10,000 U/L, and the 24-hour survival rate is 37.34%.

Furthermore, Mao et al. ^[14]^successfully constructed an EHS rat model under the conditions of (39.5 ± 0.3)°C and (55 ± 5)% RH. Following their method, we also adopted similar conditions for modeling. However, during our experiments, we observed that almost all rats could reach a core temperature of 43°C, but some did not reach exhaustion at that point, and their survival rates were much higher than 50%. We believe this difference may be attributed to variations in the rats' endurance and heat tolerance. Therefore, we established a modeling criterion based on both exhaustion and a core temperature greater than or equal to 43°C. Our observations confirmed that when the rats were exhausted, all of them had a core temperature greater than or equal to 43°C.

This improved modeling approach allows us to better reflect the clinical characteristics of EHS and provides a more accurate representation of the physiological responses in the experimental animals.

**8. In Figure 1: Annexin bars seem relatively different on the other hand your standart deviation is very high. That's why you couldn't observe statistical significance. Do you consider to repeat that data?** Thank you for your valuable feedback and suggestions. Based on your comments, it seems there might be some confusion regarding the figure number and the specific measurement. To clarify, we assume you are referring to Figure 2 instead of Figure 1.In addition, what do you mean by the "Annexin bars"? Is it caspase-3?

Regarding the high standard deviation and lack of statistical significance, we have performed our statistical analysis using appropriate software, and the presence of statistical significance was also determined using statistical software, with a p-value less than 0.05 indicating statistical difference. Our data was collected through three independent replicates and validated using multiple detection methods to ensure reliability.

We appreciate your constructive feedback and will incorporate these improvements into our revised manuscript.

**9. In Figure 4: There is no mitochondrial membrane potential bar chart. There was only two bar charts for ATP level. Please correct.**
hank you for your valuable feedback and suggestion. As requested, we have revised the manuscript to include the missing mitochondrial membrane potential bar chart alongside the two existing bar charts for ATP levels.

We appreciate your guidance in improving the clarity and completeness of our work. **10. Figure 5: There is no bar graph for ROS.**
Thank you for your valuable feedback and suggestions. As per your instructions, we have made the necessary modifications to our manuscript. Specifically, since there was no bar graph for ROS, we have now included a suitable bar graph to better illustrate our findings.

We believe these changes enhance the clarity and readability of our work. Please find the updated manuscript attached for your review. **11. There is also an increase in Bax BCL levels. How can the authors conclude that there is no apoptosis. And please also give Bax/BCL ratio**
Firstly, our in vivo experimental results indicate that apoptosis, oncosis, inflammation, and oxidative stress are all involved in the pathological mechanisms of exertional heat stroke (EHS)-related rhabdomyolysis. The observed increase in Bax and Bcl-2 levels, along with an increase in caspase-3, suggests that apoptosis is indeed an important mechanism, which does not conflict with the cell experimental findings where oncosis is identified as the primary mechanism of rhabdomyolysis.

The Bax and Bcl-2 ratio forms the "switch" for apoptosis ^[15]^. When heterodimers (Bax/Bcl-2) account for 50% or more, cells begin to undergo apoptosis^[16]^. Additionally, when homodimers of Bax exceed 80% and are appropriately induced by signals, cells will initiate apoptosis ^[16]^.

Our results show that in the EHS group, the relative content of Bcl-2 in the gastrocnemius muscle of rats is 0.23 ± 0.01, while the Bax content is 0.48 ± 0.02. This results in a Bax/Bcl-2 ratio greater than 200%, indicating that apoptosis has occurred in the EHS group.

However, it has been shown by other researchers that oncosis is dependent on both Bax and Bcl-2 but independent of caspase-3, and that the Bax:Bcl-2 ratio is a predictor of therapeutic efficacy^[5]^. Therefore, despite the presence of apoptosis as indicated by the Bax/Bcl-2 ratio, the involvement of oncosis cannot be ruled out. The coexistence of these mechanisms explains the complex pathological process of EHS-related rhabdomyolysis.

In summary, while the increase in Bax and Bcl-2 levels, along with the elevated Bax/Bcl-2 ratio, strongly indicates the occurrence of apoptosis, the concurrent presence of oncosis is supported by the fact that oncosis is also Bax- and Bcl-2-dependent but caspase-3-independent. Both mechanisms contribute to the overall pathological picture of EHS-related rhabdomyolysis. **12. The survival is very bad in EHS group. How many animals were able to finish the study? Please give “n” for each group.**
Thank you for your valuable feedback. We acknowledge that the survival rate in the EHS group was indeed low, with only 2 out of 12 rats surviving until the end of the observation period. However, to ensure comprehensive data collection, we included the gastrocnemius muscle from all rats in the study, regardless of their survival status.we ended up with only 6 animals that could complete the study across all groups. Therefore, the 'n' value for each group, including the EHS group, is 6. We have updated our figures to reflect this 'n' value accordingly. **13. Authors concluded “Therefore, we propose that the mechanism of muscle cell death in skeletal muscle cells undergoing oncosis is primarily due to ATP depletion and mitochondrial damage.” Why this alterations caused oncosis rather than apoptosis? Do authors have any suggestions for mechanism?**

Thank you for raising this insightful question regarding the mechanism behind the observed cellular response.To provide a more comprehensive explanation, we propose the following potential mechanisms:

Firstly, ATP depletion is a primary factor initiating oncosis. This rapid depletion of ATP leads to the inactivation of the Na+/K+-ATPase pump on the plasma membrane, resulting in increased intracellular sodium ([Na+]i) and chloride ([Cl-]i) concentrations. This triggers water influx, causing cellular swelling and an increase in intracellular calcium ([Ca2+]i), which ultimately results in oncosis.

Secondly, apoptosis requires ATP and the caspase system. Caspases, such as caspase-3, play a crucial role in apoptosis by cleaving poly (ADP-ribose) polymerase (PARP), a key enzyme involved in DNA repair and maintenance of genomic integrity. When DNA damage is severe, PARP activation facilitates apoptosis. However, when ATP levels are critically low, the regeneration of ADP-ribose polymers required for PARP function becomes compromised. Additionally, excessive activation of PARP can lead to the depletion of NAD+ and ATP, which are necessary for the completion of apoptosis. This energy crisis favors the progression towards oncosis instead.

In summary, the transition from apoptosis to oncosis under conditions of ATP depletion can be explained by the inability of the cell to maintain sufficient ATP levels to support the energy-demanding processes of apoptosis, such as PARP-dependent DNA repair and caspase activation. Therefore, when ATP is severely depleted, the cell resorts to oncosis as a form of non-apoptotic programmed cell death.

[1] Zhang D, Yuan R, Pan J, et al. Dihydrotanshinone Triggers Porimin-Dependent Oncosis by ROS-Mediated Mitochondrial Dysfunction in Non-Small-Cell Lung Cancer[J]. Int J Mol Sci, 2023, 24(15): 11953.

[2] Kwong S P, Huang Z, Ji L, et al. PORIMIN: The key to (+)-Usnic acid-induced liver toxicity and oncotic cell death in normal human L02 liver cells[J]. Journal of Ethnopharmacology, 2021, 270(

[3] Ma F R, Zhang C H, Prasad K V S, et al. Molecular cloning of Porimin, a novel cell surface receptor mediating oncotic cell death[J]. Proceedings of the National Academy of Sciences of the United States of America, 2001, 98(17): 9778-9783.

[4] Sun Y, Yu J, Liu X, et al. Oncosis-like cell death is induced by berberine through ERK1/2-mediated impairment of mitochondrial aerobic respiration in gliomas[J]. Biomed Pharmacother, 2018, 102(699-710.

[5] Weerasinghe P, Buja L M. Oncosis: an important non-apoptotic mode of cell death[J]. Exp Mol Pathol, 2012, 93(3): 302-308.

[6] Takeda M, Shirato I, Kobayashi M, et al. Hydrogen peroxide induces necrosis, apoptosis, oncosis and apoptotic oncosis of mouse terminal proximal straight tubule cells[J]. Nephron, 1999, 81(2): 234-238.

[7] Levin S. Apoptosis, necrosis, or oncosis: what is your diagnosis? A report from the Cell Death Nomenclature Committee of the Society of Toxicologic Pathologists[J]. Toxicol Sci, 1998, 41(2): 155-156.

[8] Giannoglou G D, Chatzizisis Y S, Misirli G. The syndrome of rhabdomyolysis: Pathophysiology and diagnosis[J]. European Journal of Internal Medicine, 2007, 18(2): 90-100.

[9] Trump B F, Berezesky I K, Chang S H, et al. The pathways of cell death: Oncosis, apoptosis, and necrosis[J]. Toxicologic Pathology, 1997, 25(1): 82-88.

[10] Majno G, Joris I. Apoptosis, oncosis, and necrosis. An overview of cell death[J]. The American journal of pathology, 1995, 146(1): 3-15.

[11] Peters A A, Jamaludin S Y N, Yapa K T D S, et al. Oncosis and apoptosis induction by activation of an overexpressed ion channel in breast cancer cells[J]. Oncogene, 2017, 36(46): 6490-6500.

[12] Trump B F, Berezesky I K. Calcium-mediated cell injury and cell death[J]. FASEB journal : official publication of the Federation of American Societies for Experimental Biology, 1995, 9(2): 219-228.

[13] He S-X, Li R, Yang H-H, et al. Optimization of a Rhabdomyolysis Model in Mice With Exertional Heat Stroke Mouse Model of EHS-Rhabdomyolysis[J]. Frontiers in Physiology, 2020, 11(16): 642.

[14] 毛汉丁, 王娇, 王宇曦, et al. 植入式生物遥测技术在劳力型热射病大鼠实验模型中的应用[J]. 解放军医学院学报, 2021, 42(10): 1068-1073+1088.

[15] 何明静, 黄世杰, 孙祥琳, et al. 熊果酸通过抑制自噬对血清剥夺诱导的H9c2细胞起抗凋亡作用[J]. 中国比较医学杂志, 2020, 30(04): 56-62.

[16] Luo Z, Li Z, Li M. The Effectiveness of Bax/Bcl-2 Gene Expression Ratio, Cardiac Function and Pulmonary Hemodynamics Indexes caused by Cardiopulmonary Rehabilitation Nursing[J]. Cell Mol Biol (Noisy-le-grand), 2022, 68(11): 83-89.
